# Remission and Relapse of Hypertension After Bariatric Surgery: A Retrospective Study on Long-Term Outcomes

**DOI:** 10.1097/AS9.0000000000000158

**Published:** 2022-04-27

**Authors:** David P. Fisher, Liyan Liu, David Arterburn, Karen J. Coleman, Anita Courcoulas, Sebastien Haneuse, Eric Johnson, Robert A. Li, Mary Kay Theis, Brianna Taylor, Heidi Fischer, Julie Cooper, Lisa J. Herrinton

**Affiliations:** From the * Kaiser Permanente Northern California, Oakland, CA; † Kaiser Permanente Washington Health Research Institute, Seattle, WA; ‡ Kaiser Permanente Southern California, Pasadena, CA; § University of Pittsburgh Medical Center, Pittsburgh, PA; ∥ Harvard T.H. Chan School of Public Health, Boston, MA.

**Keywords:** bariatric surgery, blood pressure, cohort study, hypertension, long-term outcomes

## Abstract

To compare hypertension remission and relapse after bariatric surgery compared with usual care.

**Background:**

The effect of Roux-en-Y gastric bypass and sleeve gastrectomy on hypertension remission and relapse has not been studied in large, multicenter studies over long periods and using clinical blood pressure (BP) measurements.

**Methods:**

This retrospective cohort study was set in Kaiser Permanente Washington, Northern California, and Southern California. Participants included 9432 patients with hypertension 21–65 years old who underwent bariatric surgery during 2005–2015 and 66,651 nonsurgical controls matched on an index date on study site, age, sex, race/ethnicity, body mass index, comorbidity burden, diabetes status, diastolic and systolic BP, and number of antihypertensive medications.

**Results:**

At 5 years, the unadjusted cumulative incidence of hypertension remission was 60% (95% confidence interval [CI], 58–61%) among surgery patients and 14% (95% CI, 13–14%) among controls. At 1 year, the adjusted hazard ratio for the association of bariatric surgery with hypertension remission was 10.24 (95% CI, 9.61–10.90). At 5 years, the adjusted hazard ratio was 2.10 (95% CI, 1.57–2.80). Among those who remitted, the unadjusted cumulative incidence of relapse at 5 years after remission was 54% (95% CI, 51–56%) among surgery patients and 78% (95% CI 76–79%) among controls, although the adjusted hazard ratio was not significant (hazard ratio, 0.71; 95% CI, 0.46–1.08).

**Conclusions:**

Bariatric surgery was associated with greater hypertension remission than usual care suggesting that bariatric surgery should be discussed with patients with severe obesity and hypertension. Surgical patients who experience remission should be monitored carefully for hypertension relapse.

## INTRODUCTION

Obesity is an antecedent of primary hypertension in 65–75% of cases.^1^ Since 1980, the global incidence of obesity and related comorbidities have more than doubled.^2^ Medical management of hypertension includes weight loss, lifestyle modification, and medications. Improving the success of weight loss efforts could greatly reduce the risk of hypertension with a 2003 meta-analysis of 25 randomized controlled trials showed a reduction of 1 mm Hg blood pressure (BP) for each kilogram of weight loss.^3^ However, lifestyle modifications frequently fail to generate significant sustained weight loss for most people.^4^

Roux-en-Y gastric bypass (RYGB) and sleeve gastrectomy (SG) both result in significant and sustained weight loss.^5,6^ However, the effect of these surgeries on hypertension is less clear.^7,8^ Although remission of hypertension has been observed in the short-term after bariatric surgery, few long-term studies have examined hypertension after bariatric surgery.^9^ Furthermore, some studies have relied on patient self-report of their BP rather than clinical measurements and used unclear definitions of hypertension remission and relapse.^8^ In addition, many studies have been set in small, single-institution surgery cohorts.^9^ To address these gaps in the literature, a multisite, retrospective, community-based cohort study was conducted to investigate the association between bariatric surgery and long-term remission and relapse of hypertension using BP measurements recorded in the electronic medical record.

## METHODS

### Setting

The study was set in 3 Kaiser Permanente regions: Washington (formerly Group Health), Northern California, and Southern California.^5,10–15^ Study procedures were approved by each site’s Institutional Review Board. In these regions, Kaiser Permanente provides prepaid, comprehensive, and integrated care to >8 million members. During the study period, >98% of bariatric operations were performed laparoscopically. The health systems did not provide coverage for medically supervised weight loss such as meal replacement or pharmacotherapy, resources such as group classes were provided. Patients with hypertension receive medications and monitoring.

### Study Population

The cohort study included patients 21–65 years old during January 1, 2005, through September 30, 2015, with body mass index (BMI) ≥35 kg/m^2^ who had hypertension. Each study subject was assigned an index date. For bariatric surgery patients, the index date was defined as the date of surgery. The same date was defined as the index date for individually matched nonsurgical controls. Hypertension at the index date was defined as (a) ≥2 consecutive measurements of BP ≥140/90 at least 1 week apart or (b) use of antihypertensive medication on the index date plus ≥1 outpatient hypertension diagnosis in the year before the index date. Values for BP were determined using the 2 most recent measurements before the index date. However, BP measurements during days 0–7 before the index date were not used because anticipation of surgery could have affected the patient’s BP.

Enrollment for a minimum of 12 months before the index date was required to determine exclusion criteria. Exclusion criteria included pregnancy, eclampsia or preeclampsia (International Classification of Diseases, 9th edition [ICD-9] diagnostic code 642.XX), cancer (140–209.XX), HIV (042, V08), major organ transplantation (V42.0,.1,.6,.7,.8; procedure codes 33.5, 33.6, 37.5, 41.0, 50.5, 52.8, 55.6), end-stage renal disease (V45.1; procedure codes 38.95, 39.95, 54.98), heart bypass surgery (V56.XX), respiratory failure (518.XX), and liver abscess or sequelae of chronic liver disease (572.XX). Patients with resistant hypertension, defined as 1 measurement of BP ≥140/90 mm Hg closest to the index date with ≥3 classes of antihypertensive medications or any BP level with use of ≥4 classes of medications were excluded as well, because such patients are unlikely to respond to weight loss.

After applying these exclusion criteria, patients having an RYGB or SG operation were selected for study. Other bariatric operations were not included because the health systems performed few operations such as laparoscopic adjustable banding. Bariatric operations were identified using a combination of patient registries, chart review, and procedure codes (RYGB, ICD-9 44.31, 44.38, 44.39 and Common Procedural Terminology, 4th edition 43633, 43644, 43645, 43844, 43846, 43847, S2085; SG, ICD-9 43.82 and 43.89 and Common Procedural Terminology, 4th edition 43775).

Up to 10 matched nonsurgical controls were matched to each surgical case using the following procedure. First, procedure codes were used to verify that the control patient had not had a bariatric procedure at any time during their health plan membership. Second, for each surgical patient, a pool of potential controls who were enrolled on the patient’s date of bariatric surgery were identified and assigned their index dates. Controls who met the exclusion criteria were removed from the pool. Third, matching of the potential control to the surgery patient was evaluated on the basis of study site, age, BMI (categories: 35.0–39.9, 40.0–49.9, ≥50.0 kg/m^2^), sex, race/ethnicity, Elixhauser score (≤1 unit difference between surgical and nonsurgical patients), uncontrolled BP status (BP ≥140/90; yes or no), number of distinct hypertension medication classes (0–6), and diabetes status (yes or no). Finally, the Mahalanobis distance between each surgical patient and their potential control was calculated using age, BMI, Elixhauser score, diastolic and systolic BPs, and the number of hypertension medication classes. A maximum of 10 matches were selected for each surgical patient using those with the shortest Mahalanobis distance, requiring that no control be used for >1 bariatric surgery patient.

### Data Sources and Variable Construction

As with these investigators’ previous studies, ^5,10–15^ electronic medical records, insurance claims, and other data systems were used to extract enrollment, insurance coverage, demographics, BMI, BP, medications, deaths, outpatient, inpatient, and emergency department encounters, and diagnosis and procedure codes during the study period and the year before the index date. These data were used to construct study variables (Supplemental Table 1, http://links.lww.com/AOSO/A113).

BP measurements were obtained from outpatient encounters and did not include visits to the emergency room, urgent care, or surgical outpatient settings. When multiple BP measurements were recorded on the same date, the lowest reading was used. Single-class oral BP medications were grouped in 5 categories: diuretics, calcium channel blockers, angiotensin-converting enzyme inhibitors and angiotensin II receptor blockers, alpha blockers, and beta blockers, with other combination medications counted as a sixth category.

Matching variables (age, BMI, sex, race/ethnicity, Elixhauser comorbidities, BP measurements, number of medication classes, and diabetes status) were obtained from the electronic medical record. In addition, self-reported smoking history (ever, never, unknown) at baseline was obtained from vital signs.

The outcome variable “remission of hypertension” was defined as occurring ≥125 days after the end date of the last antihypertensive medication fill followed by 2 normal BP measurements at least 7 days apart, without an elevated BP between them. The date of the second normal BP measurement was used as the date of remission. One hundred twenty-five days was used to confirm that the event was remission and not a gap in medication use, since most patients receive a 100-day supply of medication. The outcome variable “relapse of hypertension” was defined for those who went into remission as of the date that the patient either restarted antihypertensive medication or the date of the second consecutive measurement of BP ≥140/90 at least 7 days apart, without a normal BP measurement between them.

Censoring variables included disenrollment and date of death as recorded in state vital statistics files.

### Statistical Analysis

Missing values on smoking and race/ethnicity were imputed, after matching, using the predictive mean matching method.^16^ Imputation used surgery-control status (RYGB, SB, control), study site, year, age, sex, baseline BMI, systolic and diastolic BP, number of antihypertensive medication classes, Elixhauser score, and number of baseline patient, outpatient, and emergency department visits.

For modeling of remission of hypertension at 5 years, Cox proportional hazards analysis was used to estimate the hazard ratio (HR) and 95% confidence interval (CI) after adjusting for all matching variables as well as smoking history, Elixhauser score, and comorbid cardiovascular, pulmonary, renal, and mental health disease. Days since the index date was used the time axis, and follow-up started on the index date and ended on the date of outcome, or upon censoring due to the diagnosis of cancer, death, disenrollment from the health plan, the end of the study on September 30, 2015, or the end of the follow-up period, which was 5 years for the primary analysis. The proportional hazards assumption was assessed by visually inspecting a plot of the log(-log(survival)) versus the log of survival and was found to be nonproportional, therefore, step functions were used.^17^

Among those who experienced remission, relapse of hypertension at 5 years was modeled using Cox proportional hazards analysis, adjusting as described in the preceding paragraph. Days since the remission was used the time axis, and follow-up started on the date of remission and ended on the date of outcome, or upon censoring as described in the preceding paragraph.

The cumulative incidence (%) of remission and relapse at 1, 5, and 7 years was calculated using Kaplan-Meier methods. In addition, the average number of antihypertensive medication classes dispensed per person to the surgical and nonsurgical cohort during each month of the follow-up period was calculated by adding the total number of medication classes (up to 6) dispensed to each patient and dividing by the total number of patients, restricting to patients who were under observation in that month. *P* values were computed using Student *t* test. All analyses were conducted using SAS version 9.4 (Cary, NC) and R 3.4.4.

## RESULTS

The number of patients 21–65 years old during the accrual period with BMI ≥35 kg/m^2^ who underwent RYGB or SG and had ≥1 year of enrollment before their surgery date was 34,824. Of these, 12,075 (35%) had hypertension at baseline. Of those with hypertension, 493 (4%) were pregnant or had another disqualifying condition and 1932 (17%) had resistant hypertension, leaving 9650 eligible surgery patients. Of these, 9432 (98%) were successfully matched to at least 1 nonsurgical control, with the total number of controls being 66,651 (ratio, 1:7.1).

Although bariatric surgery patients and nonsurgical controls were closely matched at the time of study enrollment, surgery patients more often had a BMI ≥50 (17% vs 12%) (Supplemental Table 2, http://links.lww.com/AOSO/A113). About one-quarter of surgery patients and nonsurgical controls had systolic BP ≤139 and diastolic BP ≤89 with the use of 1 class of antihypertensive medication, and about 50% had systolic BP ≤139 and diastolic BP ≤89 with the use of 2 classes of antihypertensives. The most frequently used medications were diuretics and angiotensin-converting enzyme inhibitors/angiotensin II receptor blockers.

For the analysis of remission, among patients whose follow-up began 1 year or more before the end of study, the proportion with at least 1 year of follow-up was 92.8% among the 9432 surgery patients and 89.0% among the 66,651 controls. At 5 years, these percentages were 71.7% and 66.5% (Supplemental Table 3, http://links.lww.com/AOSO/A113). For the analysis of relapse, retention differed slightly because entry into follow-up began on the remission date and not the index date.

**FIGURE 1.**
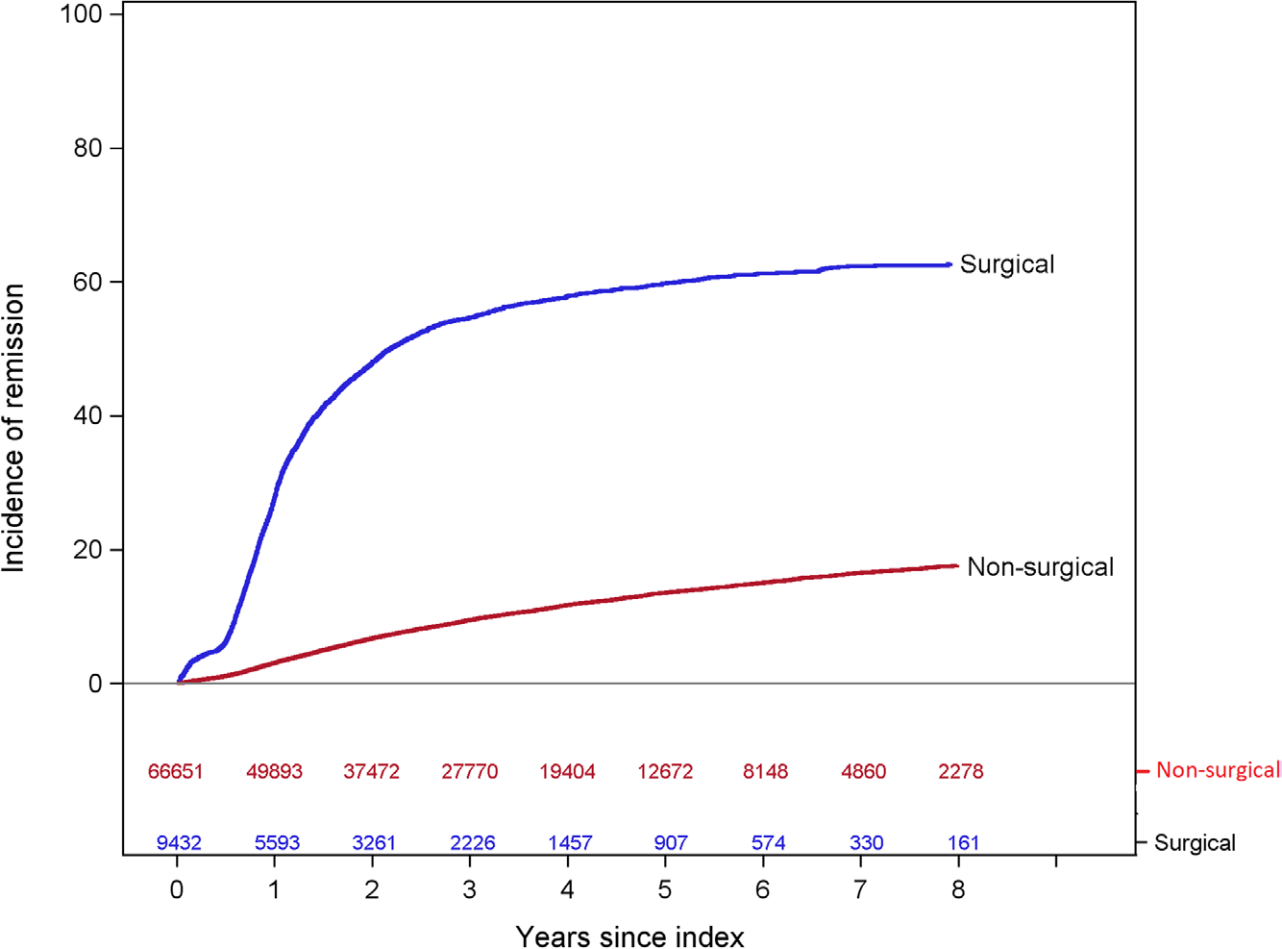
Remission: Adjusted hazard ratios and 95% CIs for the association of bariatric surgery with hypertension remission, 9432 bariatric surgery patients and 66,651 matched nonsurgical controls. (1) Hypertension was defined as ≥2 measurements of BP ≥140/90 at least 1 week apart with no nonelevated BP measures between them or use of antihypertensive medication on the date of surgery plus ≥1 outpatient hypertension diagnosis in the year before surgery. (2) For each patient who underwent bariatric surgery, up to 10 nonsurgical controls matched on the surgery/index date on site, age, sex/gender, race/ethnicity, BMI, Elixhauser score, diabetes status, uncontrolled BP status, diastolic and systolic BPs, and the number of hypertension medication classes were identified. (3) The model was adjusted for the matching variables (study site, age, BMI, sex/gender, race/ethnicity, Elixhauser score [≤1 score], uncontrolled BP status, diastolic and systolic BPs, number of distinct hypertension medication classes, and diabetes status) plus index year (continuous), smoking history, and specific physical and mental health comorbidities.

Remission of hypertension was noted in 4377 surgery patients and 5673 controls over the course of follow-up. At 1 year, the unadjusted cumulative incidence of remission was 28% (95% CI, 27–29%) among surgery patients and 3% (95% CI, 3–3%) among controls (Table 1). At 3 years, it was 55% (95% CI, 53–56%) among patients and 9% (95% CI, 9–10%) among controls. At 5 years, it was 60% (95% CI, 58–61%) among surgery patients and 14% (95% CI, 13–14%) among controls. The adjusted HR for the association of bariatric surgery with hypertension remission in the first year following the index date was 10.24 (95% CI, 9.61–10.90) (Fig. 1 and Supplemental Table 4, http://links.lww.com/AOSO/A113). The adjusted HR declined each year, but even in year 5, surgery was associated with a 2.10-fold greater incidence of remission (95% CI, 1.57–2.80).

**TABLE 1.** Cumulative Incidence (%) and 95% CI of Hypertension Remission in 9432 Bariatric Surgery Patients and 66,651 Matched Controls and Subsequent Cumulative Incidence of Hypertension Relapse Among 4377 Surgery Patients and 5673 Controls Who Had a Remission*

Outcome by HTN severity at baseline	Year 1		Year 3	
	Surgery	Control	Surgery	Control
Cumulative incidence of remission, %†				
Overall	28 (27–29)	3 (3–3)	55 (53–56)	9 (9–10)
2 drug classes	24 (23–26)	2 (2–2)	51 (49–53)	7 (6–7)
1 drug class	37 (35–39)	4 (4–4)	69 (67–71)	13 (12–13)
No drug	82 (75–88)	33 (30–36)	92 (86–96)	62 (59–66)
Cumulative incidence of relapse, %‡				
Overall	21 (20–22)	44 (43–46)	41 (40–43)	68 (66–69)
2 drug classes	23 (21–25)	51 (48–53)	45 (43–48)	74 (72–77)
1 drug class	17 (16–19)	45 (43–47)	36 (33–38)	67 (65–69)
No drug	3 (1–9)	21 (18–24)	15 (9–24)	47 (43–52)
	**Year 5**		**Year 7**	
	**Surgery**	**Control**	**Surgery**	**Control**
Cumulative incidence of remission, %‡				
Overall	60 (58–61)	14 (13–14)	62 (61–64)	17 (16–17)
2 drug classes	56 (55–58)	10 (9–10)	59 (57–61)	12 (12–13)
1 drug class	75 (73–77)	18 (17–19)	78 (75–80)	22 (21–23)
No drug	97 (90–100)	76 (72–79)	97 (90–100)	84 (80–88)
Cumulative incidence of relapse, %‡				
Overall	54 (51–56)	78 (76–79)	63 (60–66)	84 (81–86)
2 drug classes	57 (54–61)	84 (81–87)	70 (65–75)	86 (83–89)
1 drug class	48 (44–51)	78 (75–80)	54 (50–59)	86 (82–89)
No drug	30 (20–45)	58 (53–64)	37 (23–56)	65 (57–73)

*The Kaplan-Meier method was used to compute the cumulative incidence rates (%) and accounts for loss of follow-up.

†Remission was defined as 2 normal BP measures (mm Hg: ≤139 systolic and ≤89 diastolic) at least 7 days apart without an elevated BP in between the 2 measurements and after 125 days without medication.

‡For relapse, analysis was restricted to those who had a remission. Follow-up began on the date of the second normal BP after 125 days without medication as defined in the previous footnote. Relapse was defined as (a) ≥2 measurements of BP ≥140/90 at least 1 week apart, (b) with no nonelevated BP measure between the 2 measurements, and (c) no use of antihypertensive medication between the 2 measurements.

**FIGURE 2.**
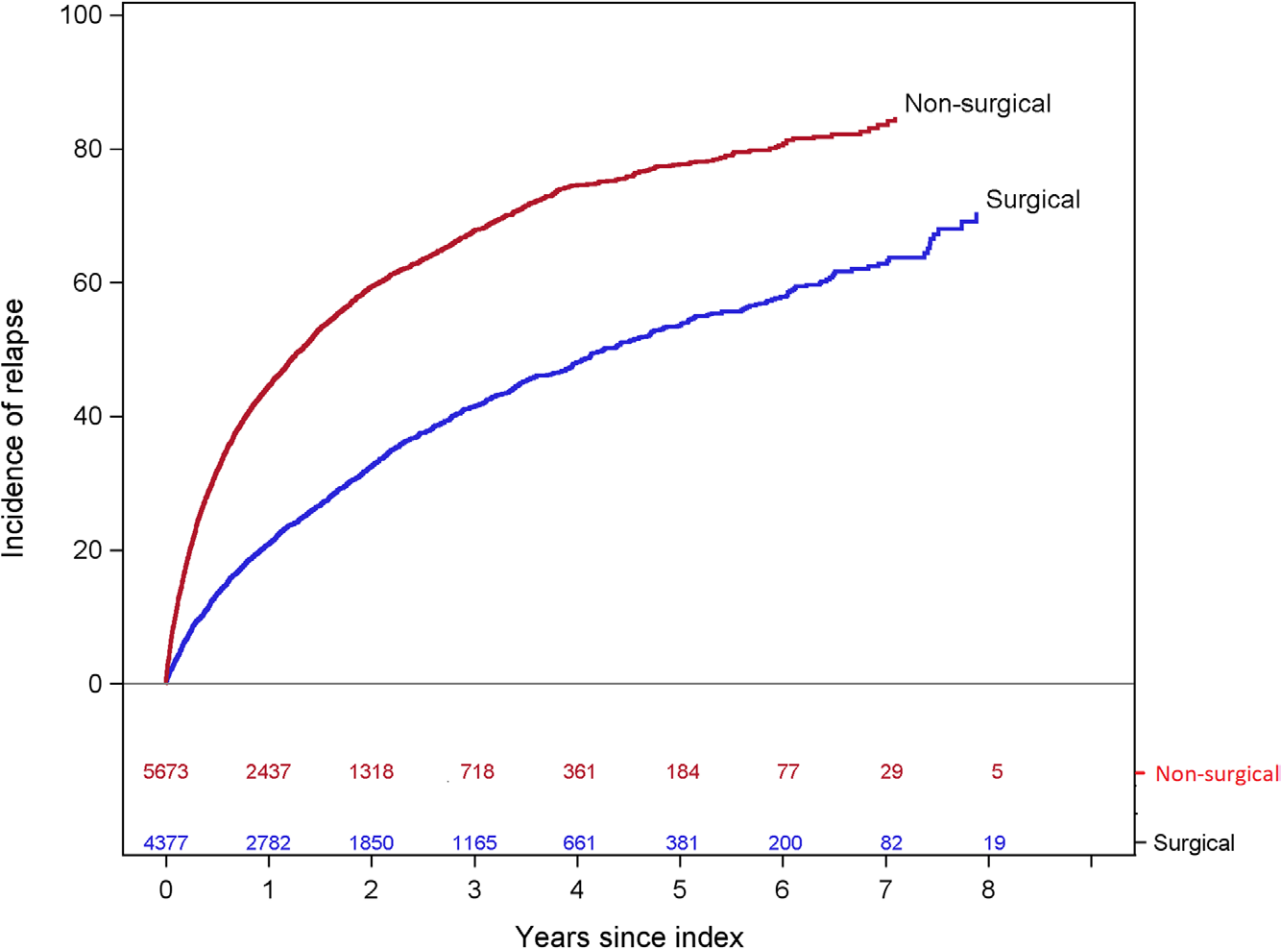
Hypertension relapse: Adjusted hazard ratios and 95% CIs for the association of bariatric surgery with relapse among those who remitted, 4377 bariatric surgery patients and 5673 matched nonsurgical controls. (1) Hypertension was defined as ≥2 measurements of BP ≥140/90 at least 1 week apart with no nonelevated BP measures between them or use of antihypertensive medication on the date of surgery plus ≥1 outpatient hypertension diagnosis in the year before surgery. (2) For each patient who underwent bariatric surgery, up to 10 nonsurgical controls matched on the surgery/index date on site, age, sex/gender, race/ethnicity, BMI, Elixhauser score, diabetes status, uncontrolled BP status, diastolic and systolic BPs, and the number of hypertension medication classes were identified. (3) The model was adjusted for the matching variables (study site, age, BMI, sex/gender, race/ethnicity, Elixhauser score [≤1 score], uncontrolled BP status, diastolic and systolic BPs, number of distinct hypertension medication classes, and diabetes status) plus index year (continuous), smoking history, and specific physical and mental health comorbidities.

Relapse was noted in 1639 of the 4377 surgery patients who remitted and 3296 of the 5673 controls who remitted. At 1 year, the unadjusted cumulative incidence of relapse was 21% (95% CI, 27–29%) among surgery patients and 44% (95% CI, 43–46%) among controls (Table 1). At 3 years, it was 41% (95% CI, 40–43%) among surgery patients and 68% (66–69%) among controls by 3 years. Among those who remitted at any time during follow-up, bariatric surgery was associated with 66% lower risk of relapse during the first year following remission (adjusted HR, 0.34; 95% CI, 0.32–0.37) (Fig. 2 and Supplemental Table 4, http://links.lww.com/AOSO/A113). This association was statistically significant through 4 years after remission but by year 5, it was no longer statistically significant (HR, 0.71; 95% CI, 0.46–1.08).

**FIGURE 3.**
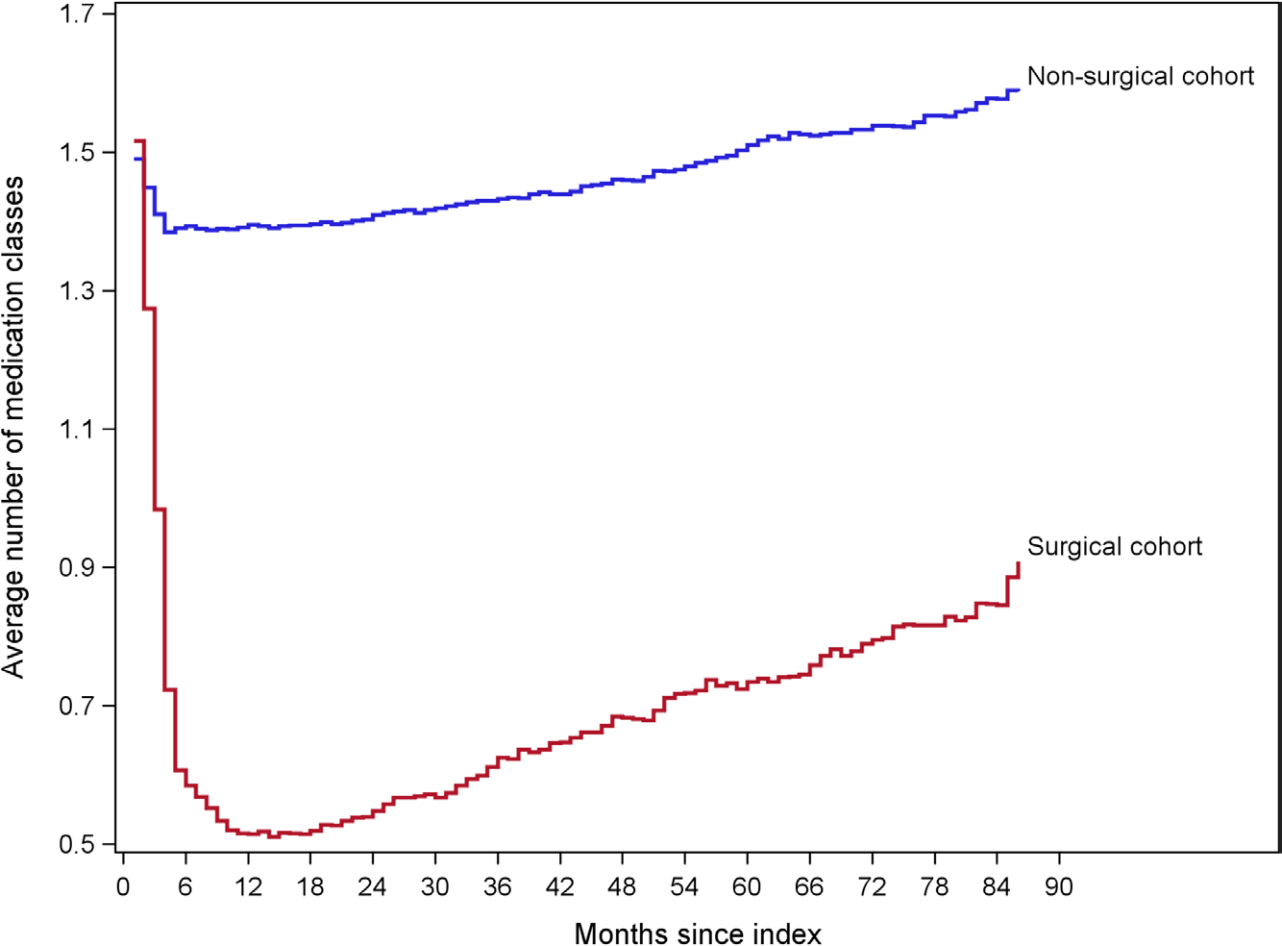
Average number of antihypertensive medication classes dispensed per patient per month. (1) Hypertension was defined as ≥2 measurements of BP ≥140/90 at least 1 week apart with no nonelevated BP measures between them or use of antihypertensive medication on the date of surgery plus ≥1 outpatient hypertension diagnosis in the year before surgery. (2) For each patient who underwent bariatric surgery, up to 10 nonsurgical controls matched on the surgery/index date on site, age, sex/gender, race/ethnicity, BMI, Elixhauser score, diabetes status, uncontrolled BP status, diastolic and systolic BPs, and the number of hypertension medication classes were identified. (3) Hypertensive medication classes included beta blocker, angiotensin II receptor antagonists, ACE inhibitors, calcium channel blocker, diuretics, and other antihypertensive medications. ACE indicates angiotensin-converting enzyme.

The average number of medication classes dispensed in the controls, per person per month, appeared to decline from 1.5 to 1.4 during the 6 months after the index date but then returned slowly to 1.5 by 5 years (Fig. 3). This contrasts (*P* < 0.0001) with the sharper decrease in the surgical group from 1.5 to 0.5 in the first year, followed by an increase to 0.7 medications by 5 years.

## DISCUSSION

In this large, retrospective cohort study of patients with severe obesity and hypertension, the 5-year unadjusted cumulative incidence of hypertension remission was 60% (95% CI, 58–61%) in 9432 bariatric surgery patients and 14% (95% CI, 13–14%) in 66,651 matched nonsurgical controls. The adjusted HR for the association of bariatric surgery with hypertension remission was 10.24 (95% CI, 9.61–10.90) in the first year and 8.85 (95% CI, 8.24–9.50) in the second year but diminished by 7 years, when the difference was no longer statistically significant. At 1 year after remission, the risk of relapse differed between surgical and nonsurgical groups, such that bariatric surgery was associated with 66% lower risk of relapse (adjusted HR, 0.34; 95% CI, 0.32–0.37). This difference narrowed after the first year but remained significant until 5 years after remission (surgical patients, 54%; controls, 78%; adjusted HR, 0.71; 95% CI, 0.41–1.08). Bariatric surgery was also associated with a striking reduction in the use of antihypertensive medications from an average of 1.5 medications per month before surgery to 0.7 per month at 5 years, while the number of medications in controls did not change appreciably. The study was novel because of its large size and 7-year duration of follow-up. The analysis of long-term changes in antihypertensive medications was novel as well, indicating a long-term benefit of surgery for reducing the need for antihypertensive medications.

These findings are also consistent with prior research. A recent systematic review found that bariatric surgery was associated with hypertension remission at 1 year ranging from 43% to 83%.^7^ Remission in this study was lower, at 28%, possibly because of the strict definition of remission, which required 125 days since the end of the last antihypertensive prescription followed by 2 consecutive normal BP readings separated by at least 1 week. This definition was used to reflect current prescribing practices in the participating health systems, which frequently issue a 100-day supply of hypertension medications to improve adherence and control. In the Swedish Obesity Study, 2-year incidence of hypertension was 62% lower in bariatric patients than controls (odds ratio, 0.38; 95% CI, 0.22–0.65)^18^ and differences in BP between surgical and control patients were larger at 2 years than at 10 years of follow-up.^19^ Hypertension control rates in the participating health systems are some of the highest reported among all health plans in the United States.^20^ This could have attenuated the differences in hypertension control between the bariatric surgery patients compared to controls given that there are aggressive efforts across all patient populations to achieve hypertension control and remission. These findings are similar to a recent clinical trial focused specifically on the 3-year hypertension outcomes of 100 patients randomized to either RYGB plus medical treatment for hypertension compared to medical treatment alone.^9^ At 1 year, half of the RYGB patients experienced remission compared with none of the medically treated patients. At 3 years, 35% of RYGB patients had continuous (ie, durable) remission compared with none of the medically treated patients. These findings are largely consistent with the results of this study at 3 years, with 55% of surgical patients experiencing a remission by 3 years and 41% of those subsequently relapsing within 3 years after remission, resulting in a 32% durable remission at 3 years.

The primary purpose of this report was to compare bariatric surgery patients to patients who did not get surgery because this is relevant to patients early in their decision-making process and addresses the question, is bariatric surgery more effective than nonsurgical treatment for improving my hypertension? Once patients have decided to consider bariatric surgery, the question then shifts towards selection the optimal procedure to improve health. To address this second questions, members of our team recently published a report comparing the effectiveness of RYGB and SG on hypertension using a subset of the data included in this analysis, of whom 1778 received RYGB and 3186 received SG, with no nonsurgical control included in the study (Reynolds). The earlier study observed no difference in the incidence of remission between the 2 bariatric procedures, although RYGB was associated with lower systolic and diastolic BP measurements over time. In both reports, nearly all remissions occurred in the first year, with some patients subsequently relapsing. In the earlier report, at 5 years, 43% of patients had remitted, although 25% relapsed, leaving 18% in remission. In the present analysis, at 5 years, 60% of surgery patients and 14% of controls remitted (adjusted HR, 2.10; 95% CI, 1.57–2.80), and among those who remitted at any time during follow-up, bariatric surgery was associated with 66% lower risk of relapse during the first year following remission (adjusted HR, 0.34; 95% CI, 0.32–0.37). In addition, the present report presents longitudinal information on use of antihypertensive drugs that was not reported in our earlier study.

In this same cohort, we previously reported on weight loss finding that RYGB patients had 28.4% total weight loss (TWL), SG 23.0%TWL, and nonsurgical patients 0.2%TWL (0.1, 0.4) at 1 year and at 5 years, RYGB had 21.7%TWL (21.5, 22.0), SG 16.0%TWL (15.4, 16.6), and nonsurgical patients 2.2%TWL (2.0, 2.5). It is likely that weight changes were the main driver in differences in the rates of hypertension (HTN) remission and relapse across surgical and nonsurgical patients. However, attribution of HTN control to weight loss is an important question, that is, beyond the scope of the current study. Understanding the mediating effect of weight loss on HTN should be the subject of future research.

Although gains in remission and relapse in bariatric surgery patients compared to controls diminished by 5 years, reduction in use of antihypertensive medications continued. This is important for reducing medication side effects. In addition, for patients who experience diabetes relapse, the benefit of bariatric surgery on microvascular complications is retained long-term, provided they remain in remission for at least 1 year.^21,22^ This could also be true for complications resulting from prolonged hypertension.

An initial drop in the number of medication classes used per month during the first 6 months of follow-up was observed in the control group. This likely was an artifact of the definition used for hypertension, which included patients who used an antihypertensive on their index date but who may have skipped days or otherwise been nonadherent to their medication during follow-up.

The control group received usual medical care that generally did not include intensive medical or lifestyle interventions for weight loss. Past studies have compared bariatric surgery to intensive medical and lifestyle interventions and reported similar results, that is, rates of hypertension remission are higher and antihypertensive medication use is lower in surgical compared with nonsurgical patients.^9,23–25^

Strengths of the study include the sample size, long follow-up, multiple study sites, and comprehensive information. The study population was diverse with 17% black and 27% Hispanic patients. Baseline hypertension, remission, and relapse were rigorously defined. The main limitation was lack of randomization. Only 1–2% of patients with severe obesity and hypertension underwent bariatric surgery, and surgical patients likely differed from controls in ways that cannot be assessed because the information was not available. Thus, the study could be biased if, at baseline, the bariatric surgery patients differed from nonsurgical controls with respect to risk factors that are related both to the decision to undergo surgery and to later remission and relapse of hypertension. It is difficult to identify a such a risk factor, particularly one that would lead to the large effects that were observed. Nonetheless, the study results should be confirmed in a randomized controlled trial.

The study also had limitations. Hypertension remission and relapse were not evaluated in relation to weight loss. Also, the patients in this study were from integrated healthcare systems that prepare patients for surgery through careful chronic disease management, possibly attenuating the effects of surgery on hypertension. Our system uses population management to assure frequent BP measurement and medication compliance, with rates of hypertension control in the general membership of 84.1% among commercial patients, 87.2% among Medicare patients, and 84.6% among Medicaid patients. Additionally, in prior research, we reported that this population is 90% commercially insured, with 6% Medicare and 4% Medicaid; thus, we had insufficient data to investigate differences in outcomes across different insurance types, and our findings may not generalize to other health care settings. Furthermore, the study excluded patients with high BP on 3 medications, and all patients on 4 medications; however, it is possible that these patients could benefit from bariatric surgery and future studies should seek to investigate this question.^26^ Finally, our study lacked resources to assess the mediating effect of weight loss and regain on hypertension remission and relapse, which should be the subject of future research.^27^

Two thirds of adults with severe obesity who seek bariatric surgery have hypertension, which is associated with an increased risk of cardiovascular disease and early mortality.^28^ In this observational comparative-effectiveness study with long-term follow-up and careful matching of surgical and nonsurgical patients, bariatric surgery was strongly associated with hypertension remission over 5 years. These findings suggest that patients with severe obesity and hypertension may benefit from bariatric surgery. Healthcare systems should discuss bariatric surgery more often as a treatment for hypertension. Notwithstanding, surgical and nonsurgical patients who experience remission should be closely monitored because of the high rate of relapse.

## Supplementary Material


