# Supplementary material for: Remission and Relapse of Hypertension After Bariatric Surgery: A Retrospective Study on Long-Term Outcomes
Source: Ann Surg Open. 2022 Apr 27;3(2):e158. doi: 10.1097/AS9.0000000000000158 (PMC10013161; doi:10.1097/AS9.0000000000000158)
Supplement: Supplementary file 1 [file as9-3-e158-s001.pdf]

Online-Only Material

1. Supplemental Table 1. Variable definitions
2. Supplemental Table 2. Baseline characteristics of hypertensive patients[a] who underwent bariatric surgery and of matched non-surgical controls, 2005-2015, %.
3. Supplemental Table 3. Availability of Follow-up Data on Bariatric and Matched Non-surgical Patients at Years 1, 5, and 7
4. Supplemental Table 4. Adjusted hazard ratios and 95% CIs for the association of bariatric surgery with remission and relapse of hypertension from Years 1-8*.**, 2005-2015
5. Supplemental Figure 1. Cumulative incidence of remission among 9,432 bariatric surgery patients and 66,651 matched non-surgical controls.
6. Supplemental Figure 2. Cumulative incidence of relapse among 4,377 bariatric surgery patients and 5,673 matched non-surgical controls who remitted.

Supplemental Table 1. Variable definitions

Exclusion Variable	ICD9 Definition
Cancer	Diagnostic code 140-209.xx
Pre-eclampsia and eclampsia	Diagnostic code 642.xx
Liver abscess & sequelae of chronic liver disease	Diagnostic code 572.xx
Heart bypass surgery	Diagnostic code V56.xx
HIV	Diagnostic code 042, V08
Lung disease	Pulmonary collapse, emphysema, edema, diagnostic code 518.xx
Organ transplant	Diagnostic codes: lung V42.6, heart V42.1, bone marrow/stem cell V42.81-.82, liver V42.7, pancreas V42.0
Cancer	Diagnostic code 140-209.xx
**Analytic Variable**	**Definition**
Diabetes[1]	Diagnostic code 250 to 250.33, 250.4 to 250.63, or 250.7 to 250.73.
Pulmonary disease	Diagnostic code for chronic respiratory failure 516, 518; for asthma 493; for shortness of breath 786; for chronic obstructive pulmonary disease 490 to 496; and for other respiratory disorders 500-508 inclusive, 510, 511, 513-516 inclusive, or 518, 519
Mental health disorders	Diagnostic code 290-319
Renal disease	Diagnostic code 189, 236, 250.4, 440, 580 to 589 inclusive, 592 to 596 inclusive, 753; or procedure code 38.95, 39.95, 54.98
Charlson/Elixhauser comorbidity index	-1, 0, 1, 2, 3, 4+
Coronary artery disease	ICD-9 diagnostic code 410, 411, 414; or ICD-9 procedure code 36.01, 36.02, 36.05, 36.06, 36.07, 36.34, 36.03, 36.10, 36.11, 36.12, 36.13, 36.14, 36.15, 36.16, 36.17, 36.19, 36.31, 36.32, 36.33; or CPT procedure code 92982, 92984, 92995, 92996, 92980, 92981, 33510, 33511, 33512, 33513, 33514, 33516, 33517, 33518, 33519, 33521, 33522, 33523, 33530, 33533, 33534, 33535, 33536, 93539, 93540.
Respiratory failure	Diagnostic codes for respiratory failure 518.51, 518.81, 997.39 or procedures of intubation 96.04, tracheostomy 31.1, 31.29, mechanical ventilation 96.7, 96.71, 96.72, 93.7, 93.9, 31.74.
Medication use	Past and concomitant use of the following medication classes: statin, other lipid-lowering, diabetic medications, antihypertensives, beta blockers, teriparitide, calcitonin, anticoagulants including warfarin and heparin, antithrombotic medications.

[1]Deyo RA, Cherkin DC, Ciol MA. Adapting a clinical comorbidity index for use with ICD-9-CM administrative databases. J Clin Epidemiol. 1992 Jun;45 (6):613-9.
[2]Inge TH, Courcoulas AP, Jenkins TM, et al.; Teen-LABS Consortium. Weight Loss and Health Status 3 Years after Bariatric Surgery in Adolescents. N Engl J Med. 2016 Jan 14;374 (2):113-23.

Supplemental Table 2. Baseline characteristics of hypertensive patients[a] who underwent bariatric surgery and of matched non-surgical controls, 2005-2015, %.

Characteristic		Bariatric surgery patients (N=9,432)	Matched non-surgical controls[b] (N=66,651)
Surgical technique	None	0	100
	Sleeve gastrectomy	43	0
	Roux-en-Y gastric bypass	57	0
Year	2005-2009	28	27
	2010-2012	42	40
	2013-2015	30	33
Site	Northern California	33	39
	Southern California	62	58
	Washington	4	4
Age, years	21-34	7	5
	35-49	42	42
	50-65	51	53
Sex/gender	Female	79	79
	Male	21	21
Race/ethnicity	African American	17	15
	Hispanic	27	28
	White	51	53
	Other	4	4
BMI, kg/m^2	35.0-39.9	33	39
	40.0-49.9	51	49
	50.0+	17	12
Elixhauser score[c]	-1	36	49
	0	33	31
	1	17	12
	2+	14	8
Comorbidity	Diabetes	46	43
	Pulmonary disease	25	22
	Renal disease	35	33
	Coronary artery disease	3	2
	Mild-to-moderate depression or anxiety	43	34
	Severe depression or anxiety	5	3
	Isolated substance abuse/eating disorder	1	1
	Bipolar, psychosis, or schizophrenia-spectrum disorders	6	6
Smoking status, self-reported	Ever	39	36
	Never	61	64
BP, mmHg, and number of drug classes			
<120 / 80			

	≥3 drug classes	5	3
	2 drug classes	10	8
	1 drug class	6	5
120-139 / 80-89			
	≥3 drug classes	11	9
	2 drug classes	24	28
	1 drug class	16	21
140-159 / 90-99			
	≥3 drug classes***	0	0
	2 drug classes	13	11
	1 drug class	9	9
	No drug	1	1
≥160 / ≥100			
	≥3 drug classes***	0	0
	2 drug classes	3	3
	1 drug class	2	2
	No drug	1	0
BP medication[d]			
	Diuretic	63	60
	ACE/ARB	68	67
	Beta-blocker	31	28
	Calcium channel blocker	14	13
	Alpha-blocker	2	2

[a] Hypertension at baseline was defined as ≥2 measurements of BP ≥140/90 at least one week apart with no non-elevated BP measures between them or use of antihypertensive medication on the date of surgery *plus* ≥1 outpatient hypertension diagnosis in the year before surgery. Patients with resistant hypertension, defined as BP ≥140/90 mmHg closest to the index date with ≥3 classes of antihypertensive medications or any BP level with use of ≥4 classes of medications were excluded.
[b] For each patient who underwent bariatric surgery, up to 10 non-surgical controls matched on the surgery/index date on site, age, sex/gender, race/ethnicity, BMI, Elixhauser score, diabetes status, uncontrolled BP status, diastolic and systolic BPs, and the number of hypertension medication classes before the surgery/index date were identified.
[c] Patients with treatment-resistant hypertension, defined as ≥140 / ≥90 on 3 ore more antihypertensive medications, were excluded.
[d] Total exceeds 100% because patients who used multiple drug classes are counted multiple times.

Supplemental Table 3. Availability of Follow-up Data on Bariatric and Matched Non-surgical Patients at Years 1, 5, and 7

	Year 1		Year 5		Year 7	
	Surgery	Control	Surgery	Control	Surgery	Control
**Outcome: Hypertension Remission****						
No. eligible for follow-up at each time point	8,456	59,032	4,599	33,666	3,344	26,209
Retention Rate, %*	92.8	89.0	71.7	66.5	63.7	60.2
Censored, %						
Disenrollment	7.0	10.7	26.2	30.7	32.1	34.9
Death	0.2	0.3	2.1	2.8	4.2	4.9
**Outcome: Hypertension Relapse*****						
No. eligible for follow-up at each time point	4,280	5,600	2,356	3,499	1,552	2,339
Retention rate, %	92.7	90.7	69.0	66.5	61.7	57.1
Censored, %						
Disenrollment	7.1	9.0	28.3	29.9	34.7	37.2
Death	0.2	0.3	2.7	3.6	3.6	5.7

*The Kaplan-Meier method was used to calculate the retention rate at each time point among patients who had not died or disenrolled from the participating health systems.

**Remission was defined as two normal BP measures (mmHg: ≤139 systolic and ≤89 diastolic) at least 7 days apart without an elevated BP in between the two measurements and after 125 days without medication.

***For relapse, analysis was restricted to those who had a remission. Follow-up began on the date of the second normal blood pressure after 125 days without medication as defined in the previous footnote. Relapse was defined as (a) ≥2 measurements of BP ≥140/90 at least one week apart, (b) with no non-elevated BP measure between the two measurements, and (c) no use of antihypertensive medication between the two measurements.

Supplemental Table 4. Adjusted hazard ratios and 95% CIs for the association of bariatric surgery with remission and relapse of hypertension from Years 1-8* ., 2005-2015**

			Model 1		Model 2	
			Remission (N=9,432/64,167)		Relapse (N=4,377/5,926)	
			HR	95% CI	HR	95% CI
**Bariatric surgery**						
	No		1.00	Ref	1.00	Ref
	Yes					
		Year 1	10.24	9.61-10.90	0.34	0.32-0.37
		Year 2	8.85	8.24-9.50	0.43	0.38-0.49
		Year 3	4.82	4.28-5.42	0.54	0.44-0.65
		Year 4	3.05	2.53-3.68	0.41	0.32-0.53
		Year 5	2.10	1.57-2.80	0.71	0.46-1.08
		Year 6	1.88	1.26-2.80	0.59	0.32-1.08
		Year 7	1.35	0.76-2.40	0.61	0.27-1.36
		Year 8	0.76	0.24-2.43	1.15	0.25-5.26
**BP uncontrolled at baseline**			2.10	1.97-2.24	0.68	0.61-0.75
**Number of antihypertensive medication classes**						
	0, 1		1.00	Ref	1.00	Ref
	2		0.52	0.50-0.54	1.20	1.13-1.27
	3		0.26	0.23-0.29	1.60	1.36-1.87
**Year**	Continuous		0.91	0.90-0.91	0.99	0.98-1.01
**Site**						
	Site A		0.70	0.63-0.76	1.32	0.13-1.54
	Site B		0.64	0.60-0.69	0.98	0.92-1.05
	Site C		1.00	Ref	1.00	Ref
**Age**						
	21-34		1.00	Ref	1.00	Ref
	35-49		0.64	0.60-0.69	1.22	1.10-1.36
	50-65		0.42	0.39-0.45	1.31	1.18-1.47
**Gender**						
	Female		1.22	1.16-1.28	1.02	0.94-1.10
	Male		1.00	Ref	1.00	Ref
**Race / ethnicity**						
	African- Am		0.77	0.73-0.82	1.62	1.49-1.76
	Hispanic		1.10	1.05-1.15	1.18	1.11-1.27
	White		1.00	Ref	1.00	Ref
	Other, unknown		0.77	0.68-0.87	1.30	1.10-1.54
**BMI**						
	35.0-39.9		1.00	Ref	1.00	Ref
	40.0-49.9		0.99	0.95-1.04	0.93	0.88-1.00
	50.0+		0.91	0.86-0.97	0.97	0.87-1.00
**Elixhauser score***						
	-1		0.87	0.83-0.91	0.94	0.87-1.00

		Model 1		Model 2	
		Remission (N=9,432/64,167)		Relapse (N=4,377/5,926)	
		HR	95% CI	HR	95% CI
	0	1.00	1.00	1.00	Ref
	1	1.02	0.96-1.08	1.15	1.06-1.26
	2+	0.85	0.79-0.93	1.31	1.17-1.46
**Physical comorbidity**					
	Diabetes	0.92	0.88-0.96	1.19	1.12-1.26
	Pulmonary disease	1.11	1.06-1.17	1.03	0.96-1.11
	Renal disease	1.16	1.11-1.21	1.06	1.00-1.12
	Coronary artery disease	0.74	0.63-0.88	1.27	1.01-1.58
**Smoking status**					
	Ever	1.03	0.99-1.08	0.99	0.93-1.05
	No self-report	0.92	0.85-1.00	0.91	0.82-1.02
	Never	1.00	Ref	1.00	Ref
**Mental health comorbidity**					
	Mild-to-moderate depression/ anxiety	1.10	1.05-1.15	1.12	1.05-1.19
	Severe depression/anxiety	1.29	1.16-1.42	1.10	0.96-1.27
	Isolated substance abuse/eating disorder	1.19	0.92-1.53	1.10	0.77-1.58
	Bipolar, psychosis, or schizophrenia-spectrum disorders	1.30	1.20-1.41	1.13	1.01-1.26

*Hypertension at baseline was defined as ≥2 measurements of BP ≥140/90 at least one week apart with no non-elevated BP measures between them or use of antihypertensive medication on the date of surgery *plus* ≥1 outpatient hypertension diagnosis in the year before surgery.

**For each patient who underwent bariatric surgery, up to 10 non-surgical controls matched on the surgery/index date on site, age, sex/gender, race/ethnicity, BMI, Elixhauser score, diabetes status, uncontrolled BP status, diastolic and systolic BPs, and the number of hypertension medication classes were identified.

***Elixhauser comorbidity score is a measure of comorbid health conditions

Supplemental Figure 1. Cumulative incidence of remission among 9,432 bariatric surgery patients and 66,651 matched non-surgical controls.

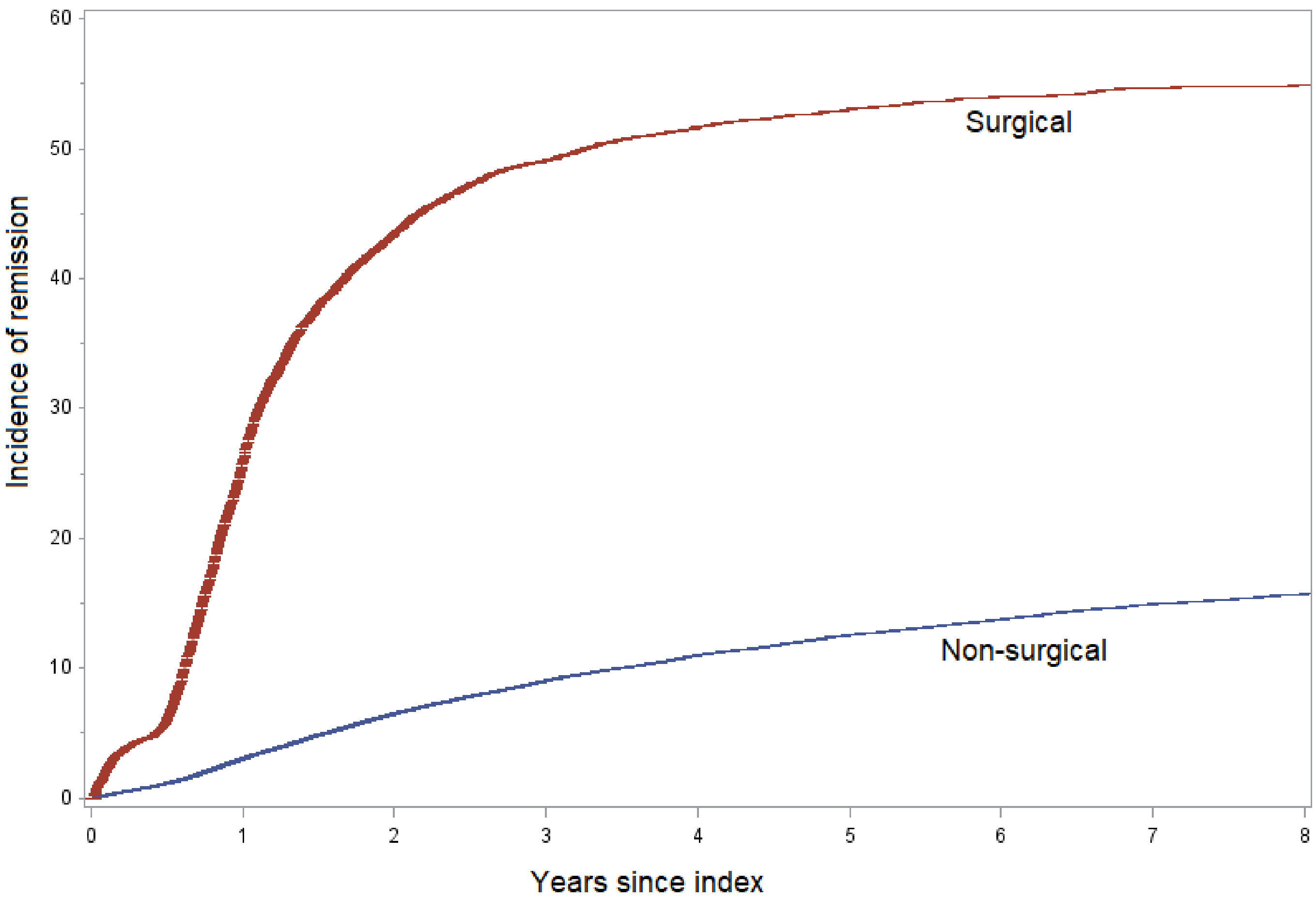

1. Remission of hypertension was defined as occurring ≥125 days past the end date of the last antihypertensive medication fill followed by two normal BP measurements at least 7 days apart, without an elevated BP in between them.
2. For each patient who underwent bariatric surgery, up to 10 non-surgical controls matched on the surgery/index date on site, age, sex/gender, race/ethnicity, BMI, Elixhauser score, diabetes status, uncontrolled BP status, diastolic and systolic BPs, number of hypertension medication classes, and number of days of health care utilization in the 7-12 months prior to the surgery/index date were identified.
3. This Kaplan-Meier analysis is matched but not otherwise adjusted.
4. The discontinuity in the curve for the surgical cohort during the first 125 days is an artifact of the definition of remission, which require 125 days off medication for patients treated with antihypertensive medications at baseline.
5. Years since the index date was used the time axis, and follow-up started on the index date and ended on the date of outcome, or on upon censoring due to the diagnosis of an disqualifying medical conditions such as cancer, death, disenrollment from the health plan, the end of the study on September 30, 2015, or the end of the follow-up period at 5 years.

Supplemental Figure 2. Cumulative incidence of relapse among 4,377 bariatric surgery patients and 5,673 matched non-surgical controls who remitted.

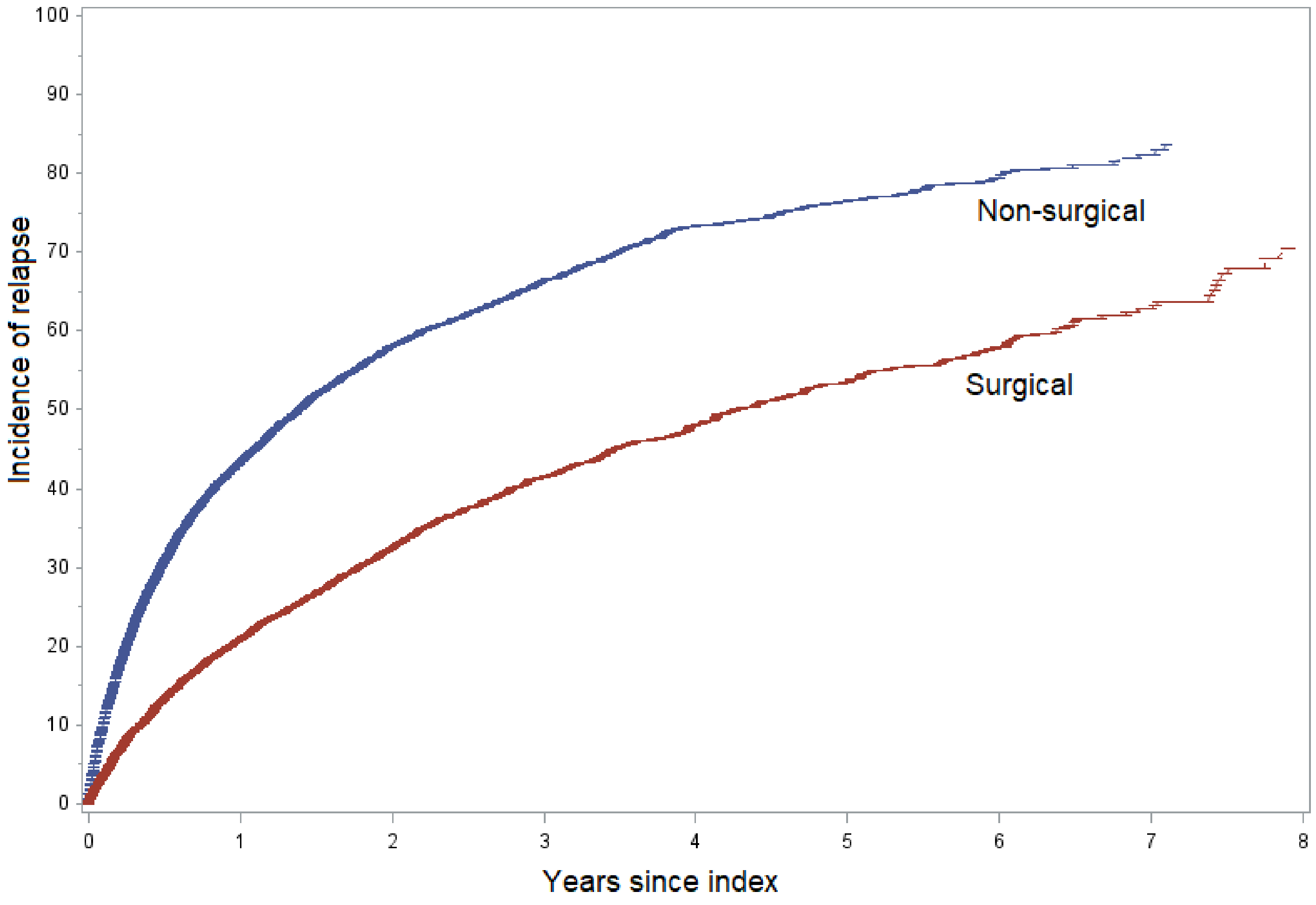

1. Hypertension relapse was defined as ≥2 measurements of BP ≥140/90 at least one week apart with no non-elevated BP measures between them or restart of antihypertensive medication.
2. For each patient who underwent bariatric surgery, up to 10 non-surgical controls matched on the surgery/index date on site, age, sex/gender, race/ethnicity, BMI, Elixhauser score, diabetes status, uncontrolled BP status, diastolic and systolic BPs, number of hypertension medication classes, and number of days of health care utilization in the 7-12 months prior to the surgery/index date were identified.
3. The analysis is matched but not otherwise adjusted.
4. The patient entered follow-up on the date of remission.
5. Years since the remission date was used the time axis, and follow-up started on the remission date and ended on the date of outcome, or on upon censoring due to the diagnosis of an disqualifying medical conditions such as cancer, death, disenrollment from the health plan, the end of the study on September 30, 2015, or the end of the follow-up period at 5 years.
